# Supplementary material for: Functional Expression of TRPV1 Ion Channel in the Canine Peripheral Blood Mononuclear Cells
Source: Int J Mol Sci. 2021 Mar 20;22(6):3177. doi: 10.3390/ijms22063177 (PMC8003907; doi:10.3390/ijms22063177)
Supplement: Supplementary file 1 [file ijms-22-03177-s001.zip › Supplementary/Tabele S1_protein accesssion numbers.docx]

Tabele 1. Accession numbers of protein sequences of the canine TRP ion channels family members, used in phylogenetic analysis.

| Nazwa Białka | Numer dostępu w bazie NCBI | Długość (aa) |
| --- | --- | --- |
| TRPV1 | NP_001003970 | 840 |
| TRPV2 | XP_025284059 | 765 |
| TRPV3 | XP_022279188 | 790 |
| TRPV4 | NP_001120787 | 871 |
| TRPV5 | XP_013975308 | 732 |
| TRPV6 | XP_013975310 | 757 |
| TRPA1 | XP_025333778 | 1118 |
| TRPM1 | XP_005618325 | 1638 |
| TRPM2 | XP_013965183 | 1602 |
| TRPM3 | XP_005615919 | 1756 |
| TRPM4 | XP_025280095 | 1211 |
| TRPM5 | XP_005631408 | 1152 |
| TRPM6 | XP_013973011 | 2040 |
| TRPM7 | XP_025328593 | 1867 |
| TRPM8 | XP_013962941 | 1104 |
| TRPC1 | [XP_013962095](https://www.ncbi.nlm.nih.gov/protein/XP_013962095.1) | 794 |
| TRPC2 | XP_013978049 | 1244 |
| TRPC3 | XP_013976956 | 941 |
| TRPC4 | XP_005635478 | 982 |
| TRPC5 | XP_013967027 | 974 |
| TRPC6 | XP_546553.3 | 932 |
| TRPC7 | XP_013973207 | 862 |
| TRPML1 | XP_025313183 | 577 |
| TRPML2 | XP_005622049 | 563 |
| TRPML3 | XP_547306.4 | 618 |
| TRPP1 | NP_001006651 | 4311 |
| TRPP2 | XP_544974.2 | 908 |
| TRPP3 | XP_013964615 | 858 |
| Kv 1.4 | XP_005633823.1 | 666 |
