# Supplementary figures and images for: Functional Expression of TRPV1 Ion Channel in the Canine Peripheral Blood Mononuclear Cells
Source: Int J Mol Sci. 2021 Mar 20;22(6):3177. doi: 10.3390/ijms22063177 (PMC8003907; doi:10.3390/ijms22063177)

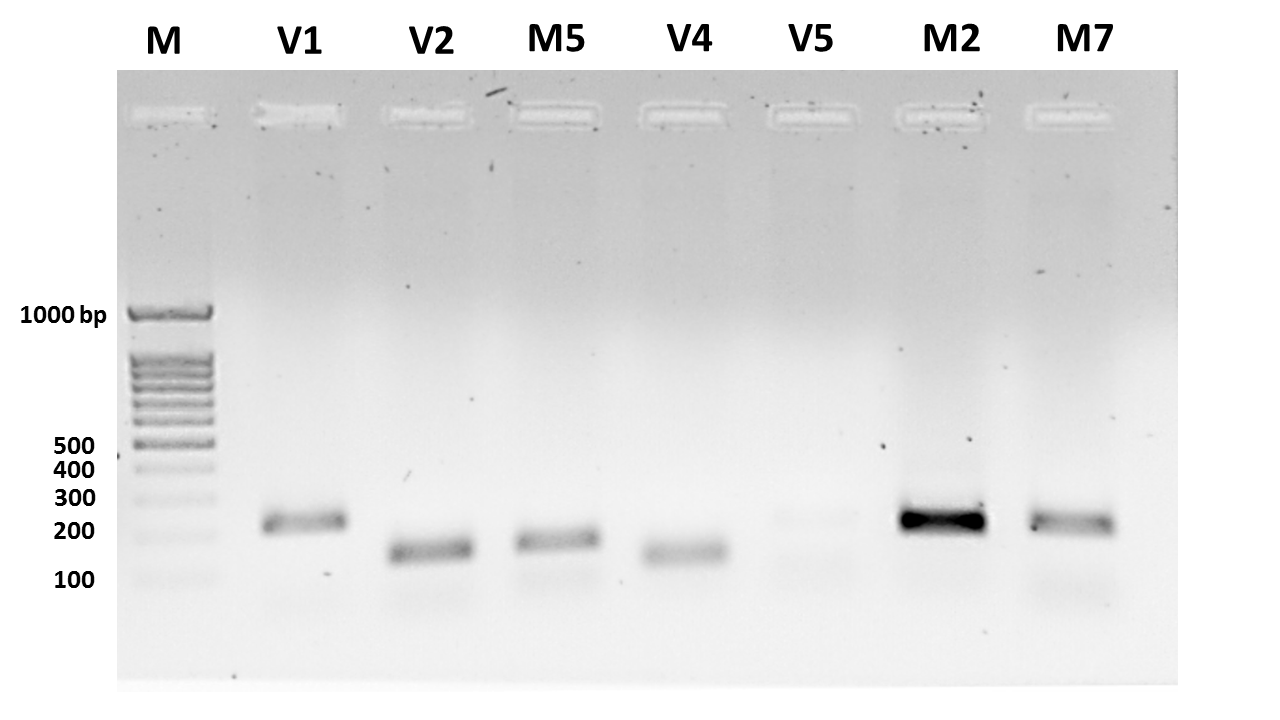

Supplement: Supplementary file 1 [file ijms-22-03177-s001.zip › Supplementary/Figure S1_gel elecrophoresis.TIF]
